# Association Analysis between g.18873C>T and g.27522G>A Genetic Polymorphisms of *OPG* and Bone Mineral Density in Chinese Postmenopausal Women

**DOI:** 10.1155/2014/320828

**Published:** 2014-12-15

**Authors:** Fei Wang, Yi Cao, Fang Li, Jianlin Shan, Tianlin Wen

**Affiliations:** ^1^Department of Orthopaedic Surgery, The General Hospital of Beijing Military Area Command, No. 5 Nanmencang Road, Dongcheng District, Beijing 100700, China; ^2^Department of Endocrinology, The General Hospital of Beijing Military Area Command, No. 5 Nanmencang Road, Dongcheng District, Beijing 100700, China

## Abstract

Several studies report that the *OPG* is an important candidate gene in the pathogenesis of osteoporosis. This study aimed to detect the potential association of *OPG* gene polymorphisms with osteoporosis in postmenopausal women. We recruited 928 subjects containing 463 with primary postmenopausal osteoporosis and 465 healthy volunteers as controls. The BMD of neck hip, lumbar spine (L_2–4_), and total hip were assessed by dual-energy X-ray absorptiometry (DEXA). Through the created restriction site-polymerase chain reaction (CRS-PCR), PCR-restriction fragment length polymorphism (PCR-RFLP), and DNA sequencing methods, the g.18873C>T and g.27522G>A have been investigated. As for g.18873C>T, our data indicated that subjects with CC genotype have significantly higher BMD value than those of CT and TT genotypes (all *P* values < 0.05). As for g.27522G>A, the BMD values of subjects with GG genotype were significantly higher than those of GA and AA genotypes (all *P* values < 0.05). Our findings suggest that the *OPG* g.18873C>T and g.27522G>A genetic polymorphisms are associated with the decreased risk for osteoporosis in Chinese postmenopausal women.

## 1. Introduction

Osteoporosis is a multifactorial disease in the postmenopausal women, which is characterized by low bone mineral density (BMD) and deteriorated microarchitecture of bone with the increased susceptibility to fracture [1–9]. BMD is a complex trait that is determined by multiple interaction of environmental, metabolic, and genetic factors [10]. It is well accepted that the genetic factors play key roles in the etiology of osteoporosis [11–16]. Growing evidence indicates that osteoprotegerin gene (*OPG*) is one of the most important candidate genes for influencing the pathogenesis of osteoporosis [9, 17–26]. Some genetic polymorphisms (such as A163G, T245G, T950C, G1181C, g.18861A>G, and g.27406C>T) in* OPG* gene have been reported to play genetic influence on BMD and osteoporosis [6, 7, 9, 10, 16, 18, 22, 25–32]. However, there are no similar related studies which reported the relationship of* OPG* g.18873C>T and g.27522G>A genetic polymorphisms with BMD and osteoporosis. Therefore, this study aims to detect these two* OPG* genetic polymorphisms and to assess their potential association with BMD and osteoporosis in postmenopausal women.

## 2. Subjects and Methods

### 2.1. Studied Subjects

In total, 928 Chinese postmenopausal women were enrolled in this case-control study, containing 463 with primary postmenopausal osteoporosis (aged 45–88 years) and 465 healthy volunteers as controls (aged 47–90 years). All subjects were recruited from the General Hospital of Beijing Military Area Command between January 2009 and November 2013. All individuals were genetically unrelated Chinese Han population and lived in Beijing, China. Those individuals suffering from present or past history of diseases or taking drugs which could affect bone metabolism were excluded from this study. This study was approved by the Ethics Committee of the General Hospital of Beijing Military Area Command. All participants have provided the informed consent for this study.

### 2.2. Measurement of BMD

The BMD of neck hip, lumbar spine (L_2–4_), and total hip were assessed through dual-energy X-ray absorptiometry (DEXA) (Norland Coopersurgical Corp., WI, USA) [33]. The value of BMD was automatically calculated from bone area (cm^2^) and bone mineral content (g) and performed as g/cm^2^.

### 2.3. DNA Extraction and Genotyping of* OPG* Genetic Polymorphisms

The peripheral venous blood was collected from each individual in this case-control study. Genomic DNA were extracted using the DNA isolation kit (Invitrogen, Carlsbad, CA, USA) and stored at −20°C until analyzed. According to the DNA sequences (GenBank ID: NG_012202.1) and mRNA sequences (GenBank ID: NM_002546.3) of the human* OPG* gene, the specific polymerase chain reaction (PCR) primers were designed by the Primer Premier 5.0 software (Premier Biosoft International, Palo Alto, CA).  Table 1 shows the sequences of primers, PCR product regions, annealing temperature, and fragment sizes. The PCR amplifications were performed on a total volume of 20 *μ*L reaction mixture containing 50 ng mixed DNA template, 1x buffer (100 mmol Tris-HCl, pH 8.3; 500 mmol KCl), 0.25 *μ*mol primers, 2.0 mmol MgCl_2_, 0.25 mmol dNTPs, and 0.5 U Taq DNA polymerase (TaKaRa, Dalian, China). The PCR protocol was carried out in an initial denaturation at 95°C for 5 min, followed by 30 cycles of 94°C for 30 s, annealing at the corresponding temperature (given in Table 1) for 30 s, and 72°C for 30 s, with a final extension at 72°C for 8 min. The genotypes of* OPG* g.18873C>T genetic polymorphism were investigated by the created restriction site-PCR (CRS-PCR) method with one of the primers containing a nucleotide mismatch, which enables the use of restriction enzymes for discriminating sequence variations [34–38]. Through the PCR-restriction fragment length polymorphism (PCR-RFLP) method, we detected the genotypes of* OPG* g.27522G>A genetic polymorphism. Following the supplier's manual, the amplified PCR products (5 *μ*L) were digested with 2 U selected restriction enzymes (performed on Table 1, MBI Fermentas, St. Leon-Rot, Germany) at 37°C for 10 h. The digested PCR products were separated by electrophoresis for 1 h at 100 V on 2.5% agarose gel including 0.5 *μ*g/mL ethidium bromide. The different genotypes were observed directly under ultraviolet (UV) light. To confirm the genotyping test results from CRS-PCR and PCR-RFLP methods, we selected random samples (10% of the total samples) to reanalyze through the DNA sequencing method (ABI3730xl DNA Analyzer, Applied Biosystems, Foster City, CA).

### 2.4. Statistical Analyses

The Hardy-Weinberg equilibrium for the genetic variants in the studied subjects was assessed by the chi-squared (*χ* ^2^) test. The distribution of allelic and genotypic frequencies was compared in the studied subjects through the chi-squared (*χ* ^2^) test. The multiple regression analyses were performed to detect the potential relationships between the variables. All data were shown as the mean ± standard deviation (SD) of the mean. The Statistical Package for Social Sciences software (SPSS, version 17.0; SPSS Inc.; Chicago, IL, USA) was utilized to evaluate all statistical analyses. Statistically significance was set at *P* value < 0.05.

## 3. Results

### 3.1. Identification and Genotyping of* OPG* Genetic Polymorphisms

Through the CRS-PCR, PCR-RFLP, and DNA sequencing methods, we have successfully detected and genotyped two* OPG* genetic polymorphisms (g.18873C>T and g.27522G>A). As for g.18873C>T, our sequence analyses indicate that this genetic polymorphism causes C→T mutation, results into a nonsynonymous mutation in exon 2 at 18873 position of* OPG* gene, and leads to threonine (Thr) to isoleucine (Ile) amino acid replacement (p.Thr20Ile, reference sequences GenBank IDs: NG_012202.1, NM_002546.3, and NP_002537.3). The PCR products of g.18873C>T were digested with* Aci*I restriction enzymes and divided into three genotypes, CC (196 bp and 20 bp), CT (216 bp, 196 bp, and 20 bp), and TT (216 bp, Table 1). As for g.27522G>A, this genetic polymorphism causes G→A mutation. It is a synonymous mutation in exon 5 at 27522 position of* OPG* gene (p. cysteine (Cys) 319Cys, reference sequences GenBank IDs: NG_012202.1, NM_002546.3, and NP_002537.3). The PCR products of 27522G>A were digested with* Sph*I restriction enzymes and divided into three genotypes, GG (173 bp and 80 bp), GA (253 bp, 173 bp, and 80 bp), and AA (253 bp, Table 1).

### 3.2. Allele and Genotype Frequencies

Table 2 shows the frequencies of allele and genotype for* OPG* g.18873C>T and g.27522G>A genetic polymorphisms. As for g.18873C>T, the genotypic frequencies in osteoporosis cases (CC, 43.41%; CT, 41.25%; TT, 15.33%) were statistically significantly different from those of healthy controls (CC, 50.97%; CT, 39.78%; TT, 9.25%; *χ* ^2^ = 9.9276, *P* = 0.0070), and significant differences were found between the allele frequencies of cases (C, 64.04%; T, 35.96%) and those of healthy controls (C, 70.86%; T, 29.14%, *χ* ^2^ = 9.8349, *P* = 0.0017). As for g.27522G>A, the allele frequencies in osteoporosis cases (G, 66.63%; A, 33.37%) were not consistent with healthy controls (G, 70.97%; A, 29.03%; *χ* ^2^ = 4.0663, *P* = 0.0437). The genotype frequencies in osteoporosis cases (GG, 46.65%; GA, 39.96%; AA, 13.39%) were significantly different from healthy controls (GG, 48.60%; GA, 44.73%; AA, 6.67%; *χ* ^2^ = 11.9014, *P* = 0.0026). As shown in Table 2, the distributions of these two genetic variants were fitted with Hardy-Weinberg equilibrium (all *P* values > 0.05).

### 3.3. *OPG* Genetic Polymorphisms Associated with BMD

The values of age, weight, height, body mass index (BMI), adjusted neck hip BMD, adjusted spine BMD, and adjusted total hip BMD in the studied subjects are shown in Table 3. Our data indicated that the* OPG* g.18873C>T and g.27522G>A genetic polymorphisms were statistically associated with the adjusted neck hip BMD, adjusted spine BMD, and adjusted total hip BMD. As for g.18873C>T, subjects with CC genotype had significantly higher adjusted BMD value than those of CT and TT genotypes (all *P* values < 0.05). As for g.27522G>A, subjects with GG genotype had significantly higher adjusted BMD value than those of GA and AA genotypes (all *P* values < 0.05).

## 4. Discussion

Osteoporosis remains an important and complex health problem in the postmenopausal women in the world. Previous studies indicated that this disease is caused by the combined effects of genetic and environmental factors [16], but the genetic factors play key roles for the development of osteoporosis [11–16]. Evidence from the published reports approved that several* OPG* genetic polymorphisms have been potentially associated with BMD and osteoporosis [6, 7, 9, 10, 16, 18, 22, 25–32]. However, findings from these observations are still inconsistent, and the exact mechanism of osteoporosis etiology is poorly understood. In this case-control study, we firstly evaluated the genetic effects of* OPG* g.18873C>T and g.27522G>A genetic polymorphisms on BMD and osteoporosis in Chinese postmenopausal women by association analyses method. Results from this study indicated that there were significant differences in the allelic and genotypic frequencies among primary postmenopausal osteoporosis patients and healthy controls (for g.18873C>T, *χ* ^2^ = 9.9276, *P* = 0.0070; for g.27522G>A, *χ* ^2^ = 4.0663, *P* = 0.0437, Table 2). Subjects with the wild genotypes of* OPG* g.18873C>T and g.27522G>A genetic polymorphisms have the significantly higher value of adjusted neck hip BMD, adjusted spine BMD, and adjusted total hip BMD than those of mutation genotypes (all *P* values < 0.05, Table 3). The allele-T of g.18873C>T and allele-A of g.27522G>A genetic polymorphisms could contribute to osteoporosis in Chinese postmenopausal women. These preliminary findings provided more evidence that* OPG* genetic polymorphisms could play genetic effects on BMD and osteoporosis. To the best of our knowledge, this is the first assessment of the influence of* OPG* g.18873C>T and g.27522G>A genetic polymorphisms with the genetic susceptibility to BMD and osteoporosis. Future replication studies on larger different populations are needed to confirm these findings from this study and to reach more reliable results for assessing the relationship of g.18873C>T and g.27522G>A or other genetic polymorphisms with the etiology of osteoporosis.

## Figures and Tables

**Table 1:** PCR, CRS-PCR, and PCR-RFLP analysis used for genotyping *OPG* SNPs.

SNPs	Primer sequences	Annealing temperature (°C)	PCR amplification fragment (bp)	Region	Restriction enzyme	Genotype method	Genotype (bp)
g.18873C>T	5′-GACATCTCCATTAAGTGGACCG-3′	58.7	216	Exon 2	*Aci*I	CRS-PCR	CC: 196, 20
	5′-GCTGCAGTATAGACACTCGTCAC-3′						CT: 216, 196, 20 TT: 216

g.27522G>A	5′-GAGCAGCTTCGTAGCTTGATG-3′	58.0	253	Exon 5	*Sph*I	PCR-RFLP	GG: 173, 80
	5′-TTGTGAAGCTGTGAAGGAACC-3′						GA: 253, 173, 80 AA: 253

Note: SNPs, single-nucleotide polymorphisms. PCR, polymerase chain reaction; PCR-RFLP, PCR-restriction fragment length polymorphism; CRS-PCR, created restriction site-PCR. Underlined nucleotides mark nucleotide mismatches enabling the use of the selected restriction enzymes for discriminating sequence variations.

**Table 2:** Genotypic and allelic frequencies of *OPG* genetic polymorphisms in the studied subjects.

Groups	g.18873C>T							g.27522G>A						
	Genotypic frequencies (%)			Allelic frequencies (%)				Genotypic frequencies (%)			Allelic frequencies (%)			
	CC	CT	TT	C	T	*χ* ^2^	*P*	GG	GA	AA	G	A	*χ* ^2^	*P*
Case group (*n* = 463)	201 (43.41)	191 (41.25)	71 (15.33)	593 (64.04)	333 (35.96)	5.0402	0.0805	216 (46.65)	185 (39.96)	62 (13.39)	617 (66.63)	309 (33.37)	4.7659	0.0923
Control group (*n* = 465)	237 (50.97)	185 (39.78)	43 (9.25)	659 (70.86)	271 (29.14)	0.6234	0.7322	226 (48.60)	208 (44.73)	31 (6.67)	660 (70.97)	270 (29.03)	3.4010	0.1826

Total (*n* = 928)	438 (47.20)	376 (40.52)	114 (12.28)	1252 (67.46)	604 (32.54)	5.5256	0.0631	442 (47.63)	393 (42.35)	93 (10.02)	1277 (68.80)	579 (31.20)	0.1689	0.9190

	*χ* ^2^ = 9.9276, *P* = 0.00700			*χ* ^2^ = 9.8349, *P* = 0.0017				*χ* ^2^ = 11.9014, *P* = 0.0026			*χ* ^2^ = 4.0663, *P* = 0.0437			

**Table 3:** Characteristics of* OPG* genetic polymorphisms in the total group of subjects.

SNPs	g.18873C>T				g.27522G>A			
Genotype	CC	CT	TT	*P*	GG	GA	AA	*P*
Number (%)	438 (47.20)	376 (40.52)	114 (12.28)	—	442 (47.63)	393 (42.35)	93 (10.02)	—
Age (years)	62.5 ± 7.8	62.7 ± 7.9	62.9 ± 6.6	0.345	61.9 ± 7.7	62.8 ± 7.9	62.9 ± 8.3	0.432
Weight (kg)	61.8 ± 6.6	62.5 ± 7.2	62.8 ± 7.2	0.236	61.5 ± 8.2	62.2 ± 6.2	62.7 ± 7.2	0.158
Height (cm)	162 ± 7.7	163 ± 6.8	165 ± 6.1	0.333	158 ± 8.5	162 ± 7.3	165 ± 6.1	0.562
BMI	23.1 ± 3.76	23.3 ± 3.55	23.5 ± 3.27	0.347	23.3 ± 3.56	23.5 ± 3.11	23.7 ± 3.26	0.256
Spine BMD (g/cm^2^)	0.931 ± 0.111	0.842 ± 0.224	0.830 ± 0.189	0.018	0.942 ± 0.102	0.856 ± 0.114	0.843 ± 0.119	0.028
Neck hip BMD (g/cm^2^)	0.752 ± 0.106	0.689 ± 0.126	0.679 ± 0.134	0.038	0.744 ± 0.176	0.682 ± 0.117	0.677 ± 0.198	0.037
Total hip BMD (g/cm^2^)	0.871 ± 0.123	0.814 ± 0.155	0.80 ± 0.187	0.042	0.869 ± 0.105	0.817 ± 0.158	0.810 ± 0.169	0.047

Note: SNPs, single-nucleotide polymorphisms; BMI, body mass index; BMD, bone mineral density; data are shown as mean ± SD (BMD values adjusted by age, weight, and height).
